# DCE-MRI radiomics models predicting the expression of radioresistant-related factors of LRP-1 and survivin in locally advanced rectal cancer

**DOI:** 10.3389/fonc.2022.881341

**Published:** 2022-08-29

**Authors:** Zhiheng Li, Huizhen Huang, Chuchu Wang, Zhenhua Zhao, Weili Ma, Dandan Wang, Haijia Mao, Fang Liu, Ye Yang, Weihuo Pan, Zengxin Lu

**Affiliations:** ^1^ Department of Radiology, Shaoxing People’s Hospital, Shaoxing Hospital, Zhejiang University School of Medicine, Shaoxing, China; ^2^ Shaoxing University School of Medicine, Shaoxing, China; ^3^ Department of Pathology, Shaoxing People’s Hospital, Shaoxing Hospital, Zhejiang University School of Medicine, Shaoxing, China; ^4^ Department of Colon and Rectal Surgery, Shaoxing People’s Hospital, Shaoxing Hospital, Zhejiang University School of Medicine, Shaoxing, China

**Keywords:** dynamic contrast-enhanced magnetic resonance imaging (DCE-MRI), locally advanced rectal cancer, radiomics models, LRP-1, survivin

## Abstract

**Objective:**

Low-density lipoprotein receptor-related protein-1 (LRP-1) and survivin are associated with radiotherapy resistance in patients with locally advanced rectal cancer (LARC). This study aimed to evaluate the value of a radiomics model based on dynamic contrast-enhanced magnetic resonance imaging (DCE-MRI) for the preoperative assessment of LRP-1 and survivin expressions in these patients.

**Methods:**

One hundred patients with pathologically confirmed LARC who underwent DCE-MRI before surgery between February 2017 and September 2021 were included in this retrospective study. DCE-MRI perfusion histogram parameters were calculated for the entire lesion using post-processing software (Omni Kinetics, G.E. Healthcare, China), with three quantitative parameter maps. LRP-1 and survivin expressions were assessed by immunohistochemical methods and patients were classified into low- and high-expression groups.

**Results:**

Four radiomics features were selected to construct the LRP-1 discrimination model. The LRP-1 predictive model achieved excellent diagnostic performance, with areas under the receiver operating curve (AUCs) of 0.853 and 0.747 in the training and validation cohorts, respectively. The other four radiomics characteristics were screened to construct the survivin predictive model, with AUCs of 0.780 and 0.800 in the training and validation cohorts, respectively. Decision curve analysis confirmed the clinical usefulness of the radiomics models.

**Conclusion:**

DCE-MRI radiomics models are particularly useful for evaluating LRP-1 and survivin expressions in patients with LARC. Our model has significant potential for the preoperative identification of patients with radiotherapy resistance and can serve as an essential reference for treatment planning.

## Introduction

Rectal cancer is the third most common malignant tumor worldwide, and approximately 70% of patients have locally advanced rectal cancer (LARC) at the initial diagnosis (1). At present, radiotherapy before surgical resection is the recommended treatment for patients with LACR (2). Preoperative radiotherapy can reduce the risk of local recurrence and ultimately improve the quality of life of patients by downstaging tumors and increasing the preservation rate of the sphincter (3). However, this effect is not ideal because the response of different individuals to preoperative radiotherapy is highly variable (4). In addition, radiotherapy is associated with long-term treatment-related toxicity, such as chronic pain, urinary incontinence, sexual dysfunction, and secondary malignant tumors (5). The identification of radioresistant LARC is a significant hurdle for patient-specific treatment.

Although the mechanism underlying radioresistance has not been fully clarified, the therapeutic effect of radiotherapy is known to depend on the cell cycle of cancer cells (6). Among all cell division phases, cells in the S, G0/G1, and G2/M phases are resistant, relatively sensitive, and sensitive to radiotherapy, respectively (7). Hence, several proteins that function in the cell cycle have been considered as potential biomarkers of radiotherapy resistance in rectal cancer. Low-density lipoprotein receptor-related protein-1 (LRP-1) is widely expressed in a wide variety of tissues; it exhibits functionalities in supporting tumor cell proliferation by promoting the entry of the cell cycle into the S phase and decreasing apoptosis (8, 9). A previous study has shown that patients with high LRP-1 expression who were subjected to radiotherapy had a poor prognosis, implying that LRP-1 could be an important marker for discriminating radioresistant rectal cancer (10). Additionally, studies have reported that survivin can also affect the radioresistance of LARC by regulating the cell cycle. Survivin is an inhibitor of apoptotic proteins that regulate both cell cycle progression and cell survival (11). According to the literature, targeted inhibition of survivin can reduce radiation-induced G2/M arrest, which means that survivin can cause more irradiation-damaged cells to enter mitosis, thus playing a vital role in radiotherapy (12). Identifying LRP-1 and survivin expressions preoperatively is helpful in making individualized treatment plans. However, the detection of LRP-1 and survivin expressions mainly depends on tissue sampling, which is limited by the invasiveness of the operation and may not reflect the entire tumor. Thus, identifying a noninvasive method for detecting LRP-1 and survivin will be beneficial and provide a reference for treatment decisions.

Radiomics analysis is an emerging field of image analysis that reflects the biological characteristics of tumors by transforming gray information into high-dimensional image features (13). Accumulating evidence indicates that radiomics can be used to quantitatively analyze tumor heterogeneity and is closely related to pathology (14). Deng et al. (15) found that radiomics predictive models have the potential to noninvasively differentiate lymph node metastasis and vascular endothelial growth factor expressions in cervical cancer. In previous studies, radiomics features have always been extracted from computed tomography angiography, T1-weighted imaging (T1WI), T2-weighted imaging (T2WI), diffusion-weighted imaging (DWI), and apparent diffusion coefficient (ADC) maps. Unlike the abovementioned imaging techniques, dynamic contrast-enhanced magnetic resonance imaging (DCE-MRI) is a relatively novel imaging modality that combines tumor morphology and changes in hemodynamics (16). DCE-MRI parameters can also reflect tumor angiogenesis and, therefore, provide essential information about the prognosis and effect of treatment for LARC (17). Moreover, in various oncology fields, several researchers have recommended using DCE-MRI to assess response to radiotherapy (18, 19). Li et al. (20) showed that radiomics features based on breast DCE-MRI could identify the status of some pathological markers (HER2 and Ki-67). Thus, radiomics analysis based on DCE-MRI might be a noninvasive method for predicting LRP-1 and survivin expressions in LARC.

This study aimed to construct and validate a noninvasive model using DCE-MRI to predict LRP-1 and survivin expressions in LARC, thus guiding clinical treatment options and improving the level of medical care.

## Materials and methods

This retrospective study was carried out in accordance with the Declaration of Helsinki and was approved by the Institutional Review Board of Shaoxing People’s Hospital. The requirement for written informed consent to review the medical records or images of the patients was waived because of the study’s retrospective nature.

### Study participants

The data of patients with pathologically confirmed LARC by biopsy or surgery from February 2017 to August 2021 at Shaoxing People’s Hospital were consecutively assessed in this study. The detailed inclusion criteria were as follows (1): histologically confirmed primary rectal adenocarcinoma (2), DCE-MRI within 2 weeks before biopsy or surgery (3), LARC determined by pretreatment MRI (≥T3 and/or N+), and (4) absence of antitumor treatment, such as neoadjuvant chemoradiotherapy, before MRI. The exclusion criteria were as follows (1): poor image quality (with severe artifacts) or failure to obtain measurements (2), severe systemic disease and absolute contraindications (3), lack of pre-surgical carcinoembryonic antigen (CEA) and carbohydrate antigen (CA)199 data (4), metastatic disease, and (5) a maximum tumor diameter of <1 cm. The standard of care for patients with LARC at our hospital was neoadjuvant chemoradiotherapy followed by total mesorectal excision (TME). However, some patients underwent TME plus adjuvant chemotherapy because of their age, risk of toxic effects, possibility of tumor progression, or painfully long treatment period. Additionally, some elderly patients chose other treatment options for the above reasons. Finally, 100 patients were enrolled in this study; among these, 64 were treated with TME plus adjuvant chemotherapy, 21 were treated with neoadjuvant chemotherapy plus TME, 12 received neoadjuvant chemoradiotherapy followed by TME, and 3 received neoadjuvant radiotherapy plus TME.

### Dynamic contrast-enhanced magnetic resonance imaging protocol

All imaging data of patients with LARC were obtained using a 3.0-T MRI scanner (Verio, Siemens, Germany) with a 12-channel phased-array body coil. Patients were required to fast for at least 8 h to empty the gastrointestinal tract and inject antispasmodic medication (Anisodamine, Minsheng, Hangzhou, China) before the MRI examination to reduce gastrointestinal artifacts. During the MRI scan, the patient was placed in the supine position, and the positioning line was located on the xiphoid process. All patients underwent a routine plain scan (T1WI, T2WI with fat suppression, T2WI, ADC, DWI) before the DCE-MRI scan and a multi-angle cross-sectional T1WI in the axial plane scan (repetition time/echo time, 3.48 ms/1.3 ms; layer thickness, 3 mm; field of view, 260 mm × 260 mm; matrix, 202 × 288; scan at multiple flip angles of 5°, 10°, and 15°). DCE-MRI adopts free breathing and is performed using a fast three-dimensional T1-weighted spoiled gradient recalled echo sequence. Multiphase dynamic enhanced scanning was performed with the following parameters: flip angle, 10°; and number of phases scanned, 35. The other acquisition parameters were the same as those above. Subsequently, a gadolinium contrast agent (Omniscan, GE Healthcare, China) was injected intravenously during phase 3 using a power injector at 0.1 mmol/kg and 3.5 mL/s. Finally, 20-mL saline was injected for flushing at the same flow rate. Contrast agent injection and data acquisition were performed simultaneously.

### Image data analysis and processing

**Figure 1:**
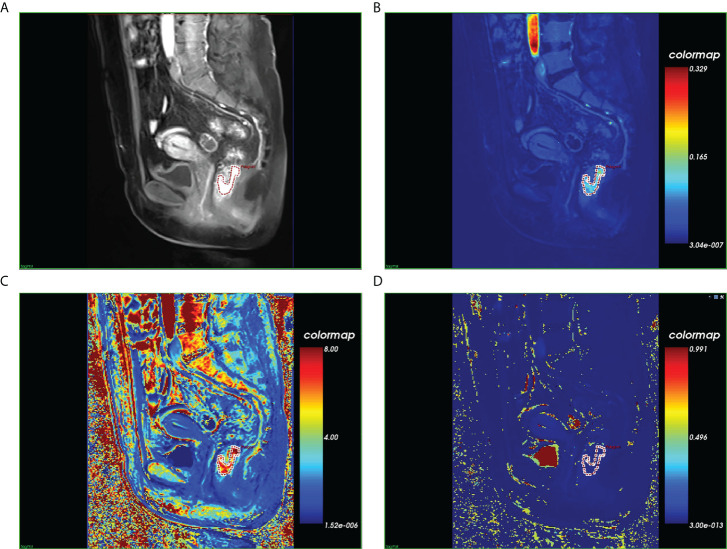
Magnetic resonance images of a histopathologically confirmed locally advanced rectal cancer in a 56-year-old woman. **(A)** Manual region of interest (ROI) placement in a contrast-enhanced sagittal T1-weighted image. **(B)** Color-coded Ktrans map of the ROI. **(C)** Color-coded Kep map of the ROI. **(D)** Color-coded Vp map of the ROI.

All sequences acquired from the DCE-MRI of eligible patients with LARC were imported into Omni Kinetics (GE Healthcare, China) software for post-processing. First, multi-flip angles of 5°, 10°, and 15° and corrected dynamic enhancement sequence scans were processed using Omni Kinetics software. Second, the external iliac artery was selected as the input artery. Third, the Tofts pharmacokinetic model was used to obtain three DCE-MRI pseudo-color images (Ktrans, Kep, and Ve). Furthermore, the region of interest (ROI) was manually delineated on each slice of the sagittal DCE pseudo-color images for calculation, using T2-weighted images as a guide. The ROI was placed in an area to avoid necrosis, calcification, and blood vessels on each slice (**Figure 1**). Two radiologists with 5 years (reader 1) and 8 years (reader 2) of specific clinical experience in rectal cancer imaging completed all image segmentations. The software automatically generated 231 radiomics features from three perfusion maps (Ktrans, Kep, and Ve), which included five categories: first-order, histogram, gray level co-occurrence matrix, Haralick, and run-length matrix.

### Immunohistochemical evaluation of low-density lipoprotein receptor-related protein-1 and survivin

LARC pathological specimens were harvested during surgery or by enteroscopy biopsy. In order to avoid the influence of anti-tumor therapy on the expressions of LRP-1 and survivin, biopsy specimens were used in patients who received any antitumor treatment before surgery. Postoperative specimens were used only in patients treated with TME plus adjuvant chemotherapy. Accordingly, there were 36 biopsy specimens and 64 surgical specimens, respectively. The expressions of LRP-1 and survivin were determined in formalin-fixed, paraffin-embedded tumor tissues using immunohistochemistry (IHC). Serial sections were immunostained with antibodies against LRP-1 (1:4000; Gene Tech, Shanghai, China) and survivin (GT204821; Gene Tech, Shanghai, China). Immunohistochemical tests for LRP-1 and survivin were performed strictly according to the IHC protocol. The expression levels of LRP-1 and survivin in tumor cells were determined using the following scoring system: 1 (≤ 10%), 2 (10% to ≤ 50%), 3 (50% to ≤ 80%), or 4 (> 80%). Staining intensity was scored as 0 (no staining), 1 (weak staining), 2 (moderate staining), or 3 (intense staining). The score for the percentage of positively stained tumor cells and the score for staining intensity were then multiplied to obtain the immunoreactive score (IRS), which ranged from 0 to 12. There was no uniform opinion on the evaluation of LRP-1 and survivin expressions on IHC, considering the limited number of patients in this study. To facilitate further statistical analyses, the expression levels of LRP-1 and survivin were divided into two categories: low expression (IRS ≤ 4 points) and high expression (IRS > 4 points). An 80% agreement between the two pathologists participating in immunostaining evaluation was set as the standard. When the pathologists disagreed regarding an evaluation, they made decisions based on consultation.

### Interobserver variability evaluation

To assess the intraobserver and interobserver reproducibilities of radiomics feature extraction, 30 patients were randomly selected and intraclass correlation coefficients (ICCs) were calculated for tumor segmentation performed 1 week later by readers 1 and 2. Subsequently, intragroup consistency analysis was performed on the features of the 30 patients drawn by reader 1, and intergroup consistency analysis was performed on the features of the same 30 patients drawn by readers 1 and 2. The reproducibility of radiomics features extracted from DCE-MRI was considered good, with intraobserver and interobserver ICC values both > 0.8. These features, with good reproducibility, were collected for subsequent radiomics analysis.

### Feature selection and radiomics signature construction

For each extracted radiomic feature, the mean value was individually subtracted from the score, which was then divided by the respective standard deviation (Z-score normalization). Subsequently, two technical approaches (the maximum relevance minimum redundancy [mRMR] method (21) combined with the least absolute shrinkage and selection operator [LASSO] method) were used to select the most useful predictive features from the training data cohort. The LASSO logistic regression model was used with penalty parameter tuning, which was conducted using tenfold cross-validation. Lambda was selected according to the 1-standard error of the minimum (1-SE) rule, where the coefficients are not rapidly changing, and the model is most parsimonious with the minimum prediction error. Multivariate logical regression was used to construct the predictive model using the selected features. A radiomics score (Rad-score) was calculated for each patient using a linear combination weighted by the respective coefficients (22).

Receiver operating characteristic (ROC) curves were used to assess the performance of the radiomics models for LRP-1 and survivin. The specificity, sensitivity, positive predictive value (PPV), negative predictive value (NPV), and area under the curve (AUC) were calculated to determine model performance. Calibration curves were used to investigate the predictive accuracy of the model graphically. The aforementioned operations were performed in both the training and validation cohorts. Finally, decision curve analysis (DCA) was used to determine the clinical usefulness of the radiomic models.

### Pathological evaluation of the therapeutic response

Tumor regression grading (TRG) was assessed in postoperative pathological specimens according to the four-tier American Joint Committee on Cancer system (23): TRG 0, no residual tumor cells; TRG 1, single cell or small group of cells; TRG 2, residual cancer with a desmoplastic response; and TRG 3, minimal evidence of tumor response. Patients were then divided into 2 groups; patients with TRG scores of 0 and 1 classified as sensitive and those with TRG scores of 2 and 3 were classified as resistant.

### Statistical analyses

All statistical analyses and figure creation were performed using R software (version 40.2; packages mainly included glmnet, pROC, rms, and rmda, Foundation for Statistical Computing, Vienna, Austria). To determine the clinical usefulness of the LRP-1 and survivin predictive model, DCA was performed by calculating the net benefits at different threshold probabilities in the test cohorts. The area under the ROC curve was calculated to measure the diagnostic efficacy of the models. Moreover, the sensitivity, specificity, PPV, NPV, and accuracy were assessed. All tests were two-tailed, and statistical significance was set at p < 0.05. Continuous variables (age) are presented as means and standard deviations, and an independent sample t-test was used to assess differences between high and low expression groups. Categorical variables (sex, location, mrT stage, mrN stage, CEA level, and CA199 level) were assessed using Fisher’s exact test or chi-squared test, as appropriate.

## Results

### Characteristics of patients with locally advanced rectal cancer

**Figure 2:**
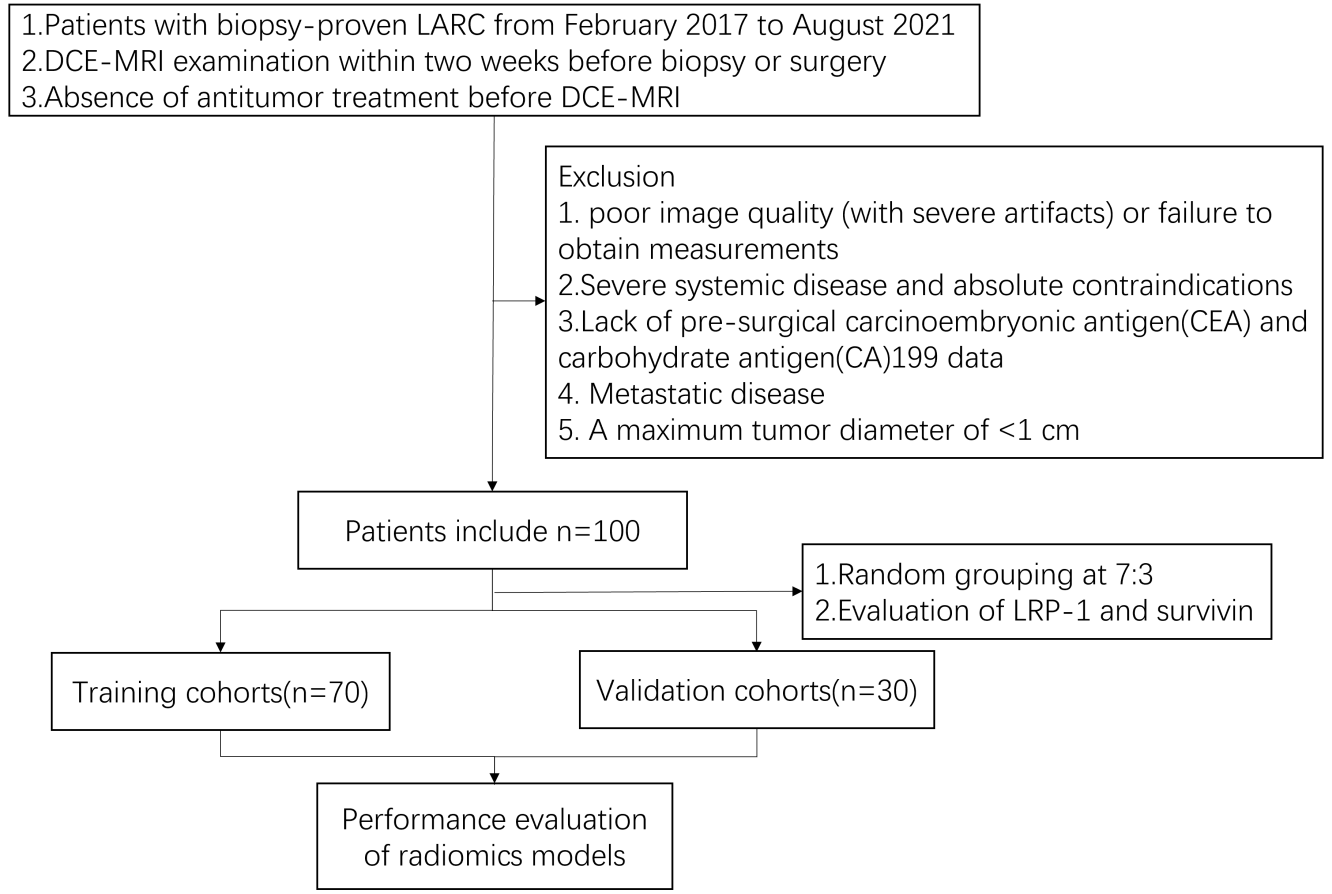
Patient inclusion and exclusion details and the patient recruitment flowchart.

This research’s summary profile is shown in **Figure 2**. Of the 100 patients, 64 were male and 36 were female. The patients were randomly divided, in a 7:3 ratio, into training (70 patients) and validation cohorts (30 patients). According to LRP-1 and survivin expression groups, no statistically significant differences were observed between the training and validation cohorts in terms of sex, age, body mass index, location, mrT stage, mrN stage, CEA level, or CA199 level (all p > 0.05). Details regarding the clinical characteristics of patients with LARC with high/low expression (LRP-1 and survivin) in both cohorts are provided in **Tables 1**, **2**.

**Table 1:** Characteristics of patients with locally advanced rectal cancer in the training and validation cohorts (low-density lipoprotein receptor-related protein-1).

Characteristic	Training cohorts(n = 70)		P	Validation cohorts(n = 30)			P
		High		Low		High		Low		
Sex			0.668				0.297	
Male	26(65.00%)	18(60.00%)		10(58.82%)	10(76.92%)			
Female	14(35.00%)	12(40.00%)		7(41.18%)	3(23.08%)			
Age(years)			0.717				0.088	
mean ± SD	68.75 ± 11.29	67.83 ± 9.13		65.47 ± 11.21	71.92 ± 7.89			
BMI(kg/m^2^)	23.45 ± 3.58	22.90 ± 3.13	0.154	23.54 ± 3.47	22.77 ± 4.06		0.088	
Location			0.254				0.237	
Above	8(26.67%)	10(25.00%)		5(29.41%)	4(30.77%)			
Straddling	15(37.50%)	6(20.00%)		5(29.41%)	8(61.54%)			
Below	15(37.50%)	16(53.33%)		7(41.18%)	1(7.69%)			
mrT stage			0.571				0.785	
T2	3(7.50%)	1(3.33%)		1(7.69%)	0(0.00%)			
T3	35(87.50%)	26(86.67%)		15(88.24%)	11(86.62%)			
T4	2(5.00%)	3(10.00%)		2(11.77%)	1(7.69%)			
mrN stage			0.843				0.758	
N0	3(7.50%)	1(3.33%)		0(0.00%)	1(7.69%)			
N1	10(25.00%)	7(23.33%)		2(11.77%)	1(7.69%)			
N2	27(67.50%)	22(73.33%)		15(88.24%)	11(84.62%)			
CEA level			0.533				0.217	
Normal	23(57.50%)	15(50.00%)		10(58.82%)	4(30.77%)			
Abnormal	17(42.50%)	15(50.00%)		7(41.18%)	9(69.23%)			
CA199 level			0.255				0.410	
Normal	32(80.00%)	27(90.00%)		15(88.24%)	10(76.92%)			
Abnormal	8(20.00%)	3(10.00%)		3(11.77%)	2(23.08%)			

**Table 2:** Characteristics of patients with locally advanced rectal cancer in the training and test groups (survivin group).

Characteristic	Training cohorts(n = 70)		P	Validation cohorts(n = 30)		P
		High		Low			High	Low	
Sex			0.353			0.193
Male	27(67.50%)	17(56.67%)		13(76.47%)	7(53.85%)	
Female	13(32.50%)	13(43.33%)		4(23.53%)	6(46.15%)	
Age(years)			0.382			0.265
mean ± SD	66.43 ± 10.71	70.93 ± 9.43		66.41 ± 9.34	22.67 ± 3.92	
BMI	23.55 ± 3.55	22.76 ± 3.14	0.339	23.61 ± 3.57	23.00 ± 3.09	0.513
Location			0.706			0.200
Above	11(27.50%)	7(23.33%)		3(17.65%)	6(46.15%)	
Straddling	13(32.50%)	8(26.67%)		6(35.29%)	2(15.38%)	
Below	16(40.00%)	15(50.00%)		8(47.06%)	5(38.46%)	
mrT stage			0.046			0.371
T2	0(0.00%)	4(13.33%)		0(0.00%)	1(7.69%)	
T3	37(92.50%)	24(80.00%)		16(94.12%)	10(76.92%)	
T4	3(7.50%)	2(6.67%)		1(5.88%)	2(15.39%)	
mrN stage			0.019			0.151
N0	0(0.00%)	4(13.33%)		0(0.00%)	1(7.69%)	
N1	8(20.00%)	9(30.00%)		3(17.65%)	0(0.00%)	
N2	32(80.00%)	17(56.67%)		14(82.35%)	12(92.31%)	
CEA level			0.268			0.127
Normal	24(60.00%)	14(46.67%)		7(69.23%)	9(41.18%)	
Abnormal	16(40.00%)	16(53.33%)		10(58.82%)	4(30.77%)	
CA199 level			0.635			0.410
Normal	33(82.50%)	26(86.67%)		15(88.24%)	10(76.92%)	
Abnormal	7(17.50%)	4(13.33%)		2(11.77%)	3(23.08%)	

### Expressions of LRP-1 and survivin

**Figure 3:**
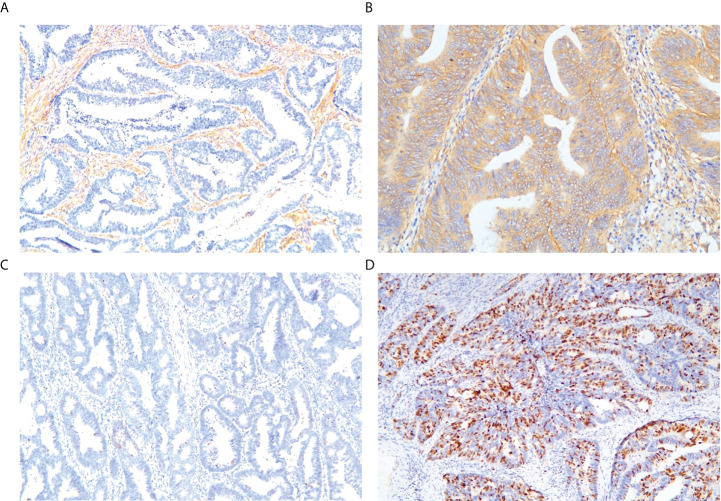
Representative immunohistochemical staining of markers. Low-density lipoprotein receptor-related protein-1: **(A)** low expression, **(B)** high expression. Survivin: **(C)** low expression, **(D)** high expression (magnification: ×10 20).

LRP-1 and survivin expressions were identified by IHC. Examples of the IHC analysis of LRP-1 and survivin expressions are shown in **Figure 3**. A weak correlation between LRP-1 and survivin was observed (r = 0.201, p = 0.045). Furthermore, some patients with high LRP expression exhibited significantly low survivin expression.

### Feature selection and radiomics model construction

**Figure 4:**
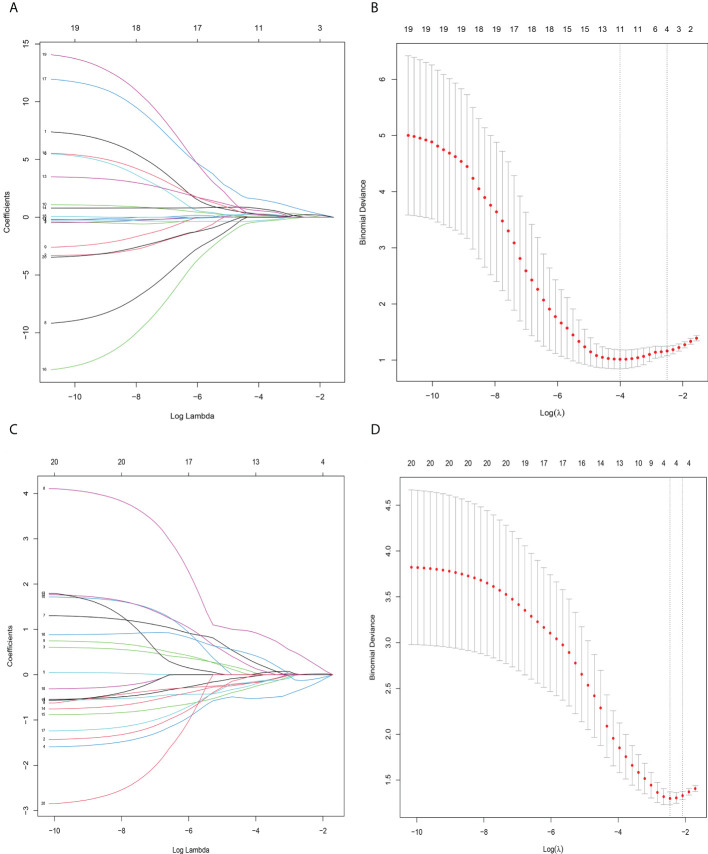
Radiomics feature selection using sthe least absolute shrinkage and selection operator (LASSO) binary logistic regression model. **(A)** LASSO coefficient profile, displaying 30 texture features. A coefficient profile plot was produced against the log (lambda) sequence. Each colored line represents the coefficient of an individual feature. **(B)** Tuning parameter (log lambda) selection in the LASSO model used tenfold cross−validation *via* 1-SE criteria. Vertical dotted lines were drawn at the selected λ values. **(A, C)** The error rate curve. **(B, D)** LASSO coefficient λ graph. Coefficient λ was selected in the LASSO using a tenfold cross-validation. We selected the coefficient λ according to the 1-SE rule.

In total, 231 features were extracted from the MRI data (67 features each from Ktrans, Kep, and Ve). The details of these selected features are provided in the **Supplemental Material**. Using the mRMR method, 20 features were identified as having high stability for predicting LRP-1 and survivin. The LASSO method with tenfold cross-validation was then used to select four potential predictive features each for LRP-1 and survivin, with non-zero coefficients, to construct the final model; the LASSO process is shown in **Figure 4**. The features were weighted according to their corresponding coefficients. The resulting Rad-score equation for the LRP-1 prediction (Rad-score1) is as follows:


“Rad−score1= 2.351 × sumAverage.Ktrans − 0.147 × skewness.Kep + 2.023 × RunLengthNonuniformity.Ve − 0.530 × differenceEntropy.Ktrans + 1.265”

The resulting Rad-score equation for the survivin prediction (Rad-score2) is as follows::


“Rad−score2 = −0.805 × differenceEntropy.Kep + 1.564 × Correlation.Ktrans − 0.069 × Quantile95.Kep − 0.236 × inverseDifferenceMoment.Ktrans + 0.565

### Radiomics model evaluation

**Figure 5:**
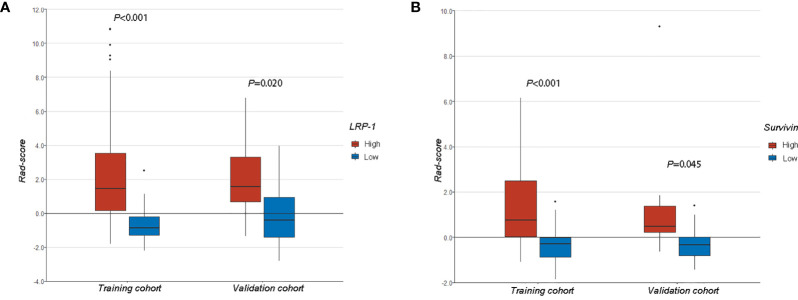
**(A)** A comparison of radiomics scores (Rad-scores) between different low-density lipoprotein receptor-related protein-1 expression levels in the training and validation cohorts. **(B)** A comparison of Rad-scores between different survivin expression levels in the training and validation cohorts.

We compared Rad-scores between the LRP/survivin (high) and LRP/survivin (low) groups in the training and validation cohorts. There was a significant difference in Rad-scores between the LRP/survivin (high) and LRP/survivin (low) groups, as shown in **Figure 5**.

**Figure 6:**
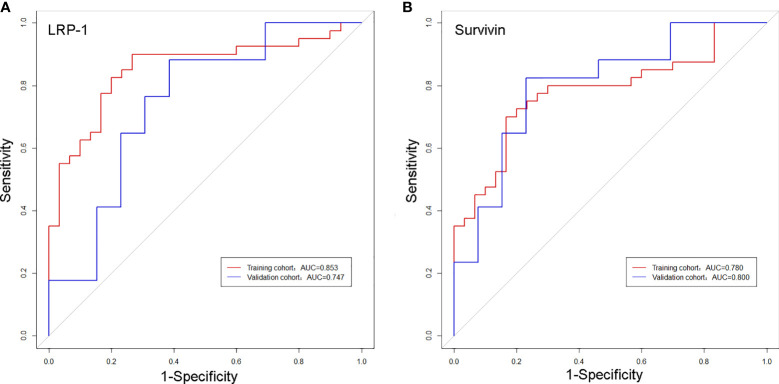
Receiver operating characteristic curves of the biomarkers for classifying low-density lipoprotein receptor-related protein-1 **(A)** and survivin **(B)** expression levels in the training and validation cohorts.

The discrimination performance of the Rad-scores for LRP-1 and survivin is summarized in **Table 3**. ROC curves of the Rad-score for predicting LRP-1 and survivin status are shown in **Figure 6**. The LRP-1 model yielded an AUC of 0.853, with a 95% confidence interval (CI), accuracy, sensitivity, and specificity of 0.760–0.945, 0.829, 0.900, and 0.733, respectively. This model was applied to the validation cohort, which generated an AUC, 95% CI, accuracy, sensitivity, and specificity of 0.747, 0.556–0.938, 0.767, 0.882, and 0.615, respectively. Correspondingly, in the training cohort, the survivin model yielded an AUC of 0.780 with 95% CI, accuracy, sensitivity, and specificity of 0.670–0.890, 0.757, 0.700, and 0.833, respectively. Analysis in the validation cohort generated an AUC, 95% CI, accuracy, sensitivity, and specificity of 0.800, 0.635–0.967, 0.800, 0.824, and 0.769, respectively.

**Table 3:** Performance summary of radiomics scores for predicting low-density lipoprotein receptor-related protein-1 and survivin status in each cohort.

	Cohort	Cut-off	AUC	ACC	SEN	SPE	PPV	NPV
LRP-1	Training	0.400	0.853	0.829	0.900	0.733	0.818	0.846
	Validation	0.445	0.747	0.767	0.882	0.615	0.750	0.800
Survivin	Training	0.557	0.780	0.757	0.700	0.833	0.848	0.676
	Validation	0.511	0.800	0.800	0.824	0.769	0.824	0.769

AUC, area under the receiver operating characteristic curve; ACC, accuracy; SEN, sensitivity; SPE, specificity; PPV, positive predictive value; NPV, negative predictive value.

**Figure 7:**
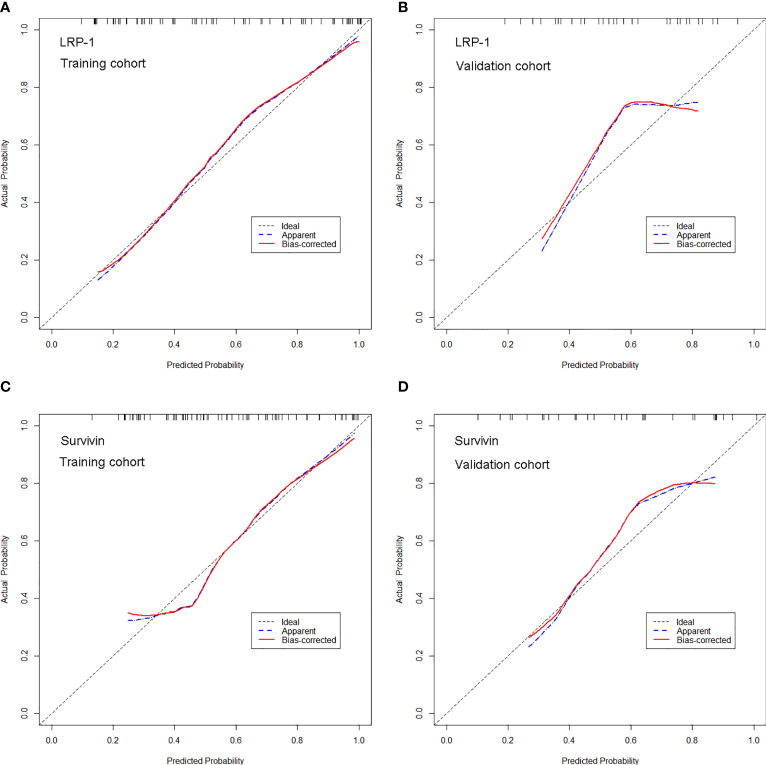
Calibration curves of the radiomics model for predicting low-density lipoprotein receptor-related protein-1 (LRP-1) and survivin expression levels in the training and validation cohorts. **(A, B)**. Calibration curves of the model for LRP-1 in **(A)** the training and **(B)** validation cohorts. **(C, D)** Calibration curves of the model for survivin in **(C)** the training and **(D)** validation cohorts. In the calibration plots, the 45° line represents marks the location of the ideal model. The blue line represents the predicted performance of the model, and the red line represents the bias correction in the model.

**Figure 8:**
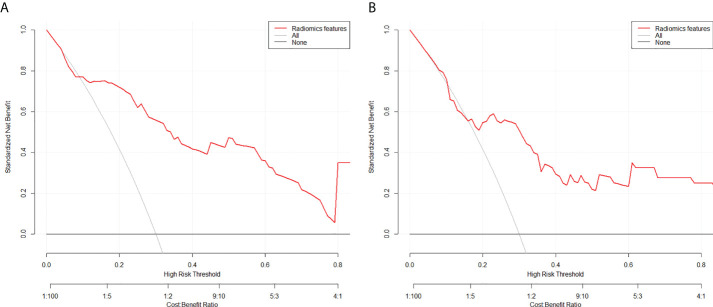
Decision curve analysis for the model for **(A)** low-density lipoprotein receptor-related protein-1 and **(B)** survivin in the test cohorts. The y-axis measures the standardized net benefit. The red curve represents the radiomics model. The gray curve represents the assumption that all patients were treated, and the straight black line at the bottom of the figure represents the assumption that no patient was treated.

The calibration curves of the two models demonstrated that the predicted probability fit well with the actual expression levels in both the training and validation cohorts, indicating excellent calibration of the radiomics models (**Figure 7**). The decision curve of the radiomics model is shown in **Figure 8**, and DCA revealed that using the radiomics models (LRP-1/survivin) added more net benefit than the treat-all or treat-none strategies, indicating the excellent performance of the radiomics models in terms of clinical application.

### Rad-scores in resistant and sensitive groups

Among 15 patients who underwent radiotherapy (12 received neoadjuvant chemoradiotherapy followed by TME and 3 received neoadjuvant radiotherapy plus TME), Rad-score2 was not significantly different between sensitive and resistant groups (p = 0.594; **Table 4**). However, Rad-score1 was significantly higher in the resistant group (4.829 ± 3.459) than in the sensitive group (-0.210 ± 0.648, p = 0.043; **Table 4**).

**Table 4:** Radiomics scores between resistant and sensitive groups.

	Sensitive groups(n=10)	Resistant groups(n=5)	F value	P value
Rad-score1	-0.210 ± 0.648	4.829 ± 3.459	2.891	0.043
Rad-score2	1.771 ± 0.415	-0.083 ± 0.947	-0.547	0.594

## Discussion

To the best of our knowledge, this study is the first attempt to propose and validate noninvasive radiomics models based on DCE-MRI for the preoperative prediction of LRP-1 and survivin expressions in patients with LARC. The predictive model for LRP-1 demonstrated favorable discrimination and yielded AUCs of 0.853 and 0.747 in the training and validation groups, respectively. In predicting survivin expression, the radiomics model achieved AUCs of 0.780 and 0.800 in the training and validation cohorts, respectively. The results suggest that the clinical use of radiomics is promising in terms of the preoperative prediction of LRP-1 and survivin. In addition, Rad-score1 was significantly higher in the resistant group than in the sensitive group, although Rad-score2 was not significantly different between the two groups. Thus, the predictive models may be helpful in guiding clinicians in identifying patients who are radiotherapy resistant and selecting appropriate treatment plans for patients with LARC.

In recent years, radioresistance has mainly been responsible for treatment failure and mortality in patients with LARC receiving radiation therapy. Furthermore, the current standard of care for LARC is to apply the same treatments to all patients, regardless of their individual responses to radiotherapy. This uniform treatment method inevitably leads to undertreatment or overtreatment for several patients with LARC (24). Currently, approximately 30%–50% of patients are reported to show radioresistance to ionizing radiation (IR); however, if these patients are identified before surgery, more intensive chemotherapy could be applied (25). In contrast, the complete response for tumors predicted to undergo invasive radical surgery may even be modified. Therefore, the development of new biomarkers capable of successfully assessing patients’ radio-responsiveness status preoperatively is urgently needed to establish patient-specific treatment (26). Radioresistance is a complex process involving the alteration of several cellular mechanisms (27). Moreover, cell division phases profoundly influence the response to radiation in cancer (28, 29). Numerous studies have shown that LRP-1 and survivin play essential roles in regulating the cell cycle, which is significantly related to radiotherapy tolerance in LARC. Identifying LRP-1 and survivin expressions before surgery may improve prognostication and guide the selection of a clinical treatment plan. At present, a pathological examination is the gold standard for diagnosing LRP-1 and survivin expressions. However, results are influenced by sampling and may be inadequately comprehensive because of tumor heterogeneity. With the advent of the precision medicine era, single-modality medical imaging is gradually evolving and cannot meet the requirements of individualized treatment (30, 31). Radiomics, an emerging technique in computational medical imaging, can extract information-rich imaging functions with high throughput and quantify imaging information that the human eye cannot detect (32, 33). Many prior studies have shown that radiomics can effectively predict the expression of multiple pathological biomarkers in various tumors based on quantitative image features derived from different MRI techniques (34, 35). In our study, we used DCE-MRI to construct a predictive model. Unlike conventional MRI imaging techniques, DCE-MRI has the advantage of estimating blood flow, blood volume, and vascular permeability and the tumor vascular microenvironment. DCE-MRI has been applied in several tumor studies and has yielded satisfactory results (36). However, no study has aimed to determine the expressions of LRP-1 and survivin. In view of this knowledge gap, we established radiomics models based on DCE-MRI to distinguish between LRP-1 and survivin expression levels, and obtained promising results.

LRP-1 is a multifunctional scavenger receptor that belongs to the low-density lipoprotein receptor family (37). Owing to its capacity to control the pericellular levels of various growth factors and proteases, LRP-1 plays a crucial role in tumor progression. Compared to untreated cells with LRP-1 inhibition, treated cells present an increase in the proportion of cells in the G1 phase and a decrease in the S phase cell population (8). Clinically, high LRP-1 expression in patients subjected to radiotherapy has a poor prognosis. Certainly, LRP-1 expression status has a crucial role in predicting radiotherapy resistance and prognosis in patients with LARC; however, few studies have used radiomics features extracted from pretreatment DCE-MRI to predict LRP-1 expression. In this study, four DCE-MRI radiomics features (Ktran. sum Average, Ktrans. difference entropy, Kep. skewness, and Ve. run length nonuniformity) were selected to construct the predictive model for LRP-1, which yielded a high AUC in both training (AUC = 0.853) and validation (AUC = 0.747) cohorts. Moreover, features from Ktrans were most commonly used in the optimal radiomics model (2/4). Previous studies have corroborated that Ktrans reflects vessel blood flow and is the product of vessel permeability and vessel surface area (38). Theoretically, the value of Ktrans is mainly determined by blood flow or elevated vessel permeability. Devy et al. (39) revealed that LRP-1 also plays an essential role in the angiogenic processes for tumor growth through its wide spectrum of interactions. Thus, the observed association between Ktrans (sum average) and LRP-1 expression is reasonable. Our radiomics model may serve as a novel quantitative tool for individually predicting the expression of LRP-1 and selecting appropriate targeted therapies for patients with LARC.

In addition, we constructed a radiomics model to predict the expression of survivin and achieved excellent results, with sensitivities of 70.0% and 76.5%, specificities of 83.3% and 69.2%, and AUCs of 0.780 and 0.800 in the training and validation cohorts, respectively. Notably, the performance of the radiomics model in the test cohort was superior to that in the validation cohort (AUC, 0.800 vs. 0.780), which demonstrates the robustness of our model. Survivin is a unique member of the inhibitor of apoptosis protein family that is expressed in most cancer cells but is barely detected in most normal adult tissues (40). Without doubt, survivin has attracted a great deal of interest as an anti-radiotherapy factor, and its overexpression in tumors has been shown to be associated with radioresistance, poor prognosis, and drug resistance (41, 42). Previous studies have shown that targeted inhibition of survivin in cancer cells can interfere with their DNA repair ability and increase their radiosensitivity to IR (12, 42). Two features from Ktrans were selected in the radiomics model for predicting survivin. In several studies, Ktrans has been considered as a robust and clinically useful biomarker of radiation resistance in some tumor types (43). These results demonstrate that Ktrans plays an essential role in reflecting the expression levels of LRP-1 and survivin. Our findings also highlighted a weak correlation between LRP-1 expression and survival (r = 0.201, p = 0.045). This correlation between LRP-1 and survivin suggests that they may play a synergistic role in radiotherapy resistance in LARC to some extent. LRP-1 or survivin may have a specific association with radiotherapy tolerance in rectal cancer, and targeted inhibition of LRP-1 or survivin can improve the prognosis of patients. However, as a puzzling part of our study, we found that some patients with high LRP-1 expression exhibited low survivin expression. These results are entirely contrary to our initial conjecture that the presentation of LRP-1 should be consistent with that of survivin to a certain extent. In our view, this inconsistency may originate from the heterogeneity of the tumor and the complex mechanisms of IR. Radiotherapy tolerance is a complex process involving many mechanisms. The biological behavior of LARC radioresistance may be reflected by multiple biomarkers rather than a single biomarker (LRP-1 or survivin). In the future, with further research on radiotherapy tolerance mechanisms, the inclusion of more tolerance factors may help improve the predictive model for radiotherapy tolerance. Further studies with larger sample sizes are required to investigate the clinical validation and additive values of the radiomics model for predicting the response to radiotherapy.

Furthermore, the calibration curve of the predictive radiomics models demonstrated good agreement between the predicted and actual probabilities in the training and validation cohorts, indicating that our models accurately evaluated the true values of LRP-1 and survivin expression. DCA showed a higher overall net benefit with the radiomics model, thus highlighting its value as an excellent tool, based on DCE-MRI, for assistance in clinical decision-making. Using the radiomics model, if a patient is predicted to have high LRP-1 or survivin expression, the administration of targeted therapy or more intensive chemotherapy should be recommended. In the future, patients with high LRP-1 and survivin-expressing LARC may serve as an ideal population for testing newer therapies.

**Figure 9:**
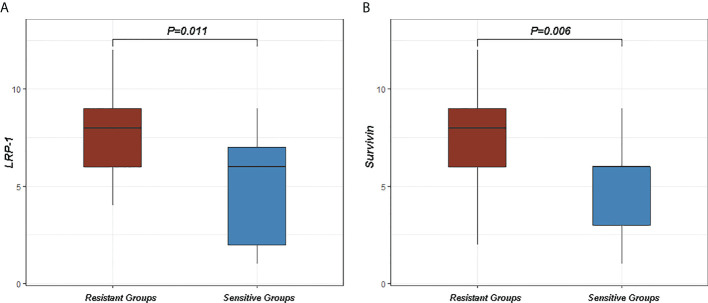
Differences in low-density lipoprotein receptor-related protein-1 (LRP-1) and survivin between sensitive and resistant groups. **(A)** The expression of LRP-1 was higher in the resistant group than in the sensitive group (p = 0.011). **(B)** The expression of survivin was higher in the resistant group than in the sensitive group (p = 0.006).

In order to investigate the relationship between LRP-1 and survivin expression and resistance to neoadjuvant radiotherapy in patients with LARC, we collected additional LARC biopsy specimens from 27 patients who received neoadjuvant radiotherapy with/without DCE-MRI examinations from February 2017 to August 2021 at Shaoxing People’s Hospital; these patients were not among the 100 patients in the main analyses. LRP-1 expression was significantly higher in the resistant group than in the sensitive group (7.813 ± 2.297 vs 5.000 ± 2.828, p = 0.011). In addition, survivin expression was significantly higher in the resistant group than in the sensitive group (7.500 ± 2.318 vs 4.636 ± 2.385, p = 0.006), as shown in **Figure 9**. These results further suggest that LRP-1 and survivin may be predictive markers clinically relevant to resistance to neoadjuvant radiotherapy in patients with LARC.

Regarding whether the effects of radiation therapy can be predicted using radiomics models, we find that although radiomics models based on DCE-MRI performed well, the present study has some limitations. First, this was a retrospective study from a single institution, which may lead to potential selection biases; also, the predictive model was not tested with external test data. Therefore, prospective and multicenter studies are encouraged in the future. Second, the number of patients was relatively small; therefore, it is necessary to incorporate more cases in future studies to determine the proposed model’s reliability. Third, additional pathological, clinical, and radiological characteristics were not considered in our study. Finally, the present study was merely based on DCE-MRI, and several previous studies have shown that the combined application of more MRI sequences (e.g., T2WI, T1WI, and DWI) may improve the predictive ability of the radiomics model. Despite these limitations, the application of the radiomics models may have clinical prospects in terms of precision and personalized medicine for patients with LARC.

## Conclusion

In conclusion, the present study demonstrated that radiomics analysis of DCE-MRI features facilitates the determination of LRP-1 and survivin expression levels in LARC before treatment. Our models have significant potential for the preoperative identification of patients with radiotherapy resistance and can serve as an essential reference for treatment planning.

## Data availability statement

The original contributions presented in the study are included in the article/**Supplementary Material**. Further inquiries can be directed to the corresponding author.

## Ethics statement

Ethical review and approval was not required for the study on human participants in accordance with the local legislation and institutional requirements. Written informed consent for participation was not required for this study in accordance with the national legislation and the institutional requirements.

## Author contributions

ZXL and ZZ designed the study and helped to revise the manuscript. ZHL was involved in the study design, analyzed and interpreted the patient data regarding locally advanced rectal cancer, performed some image processing, and was the major contributor to the writing of the paper. CW, HM, DW, and WP collected the clinical data. HH, CW, YY, and FL performed the immunohistochemical analysis. All authors have read and approved the final manuscript.

## Funding

This work was supported by the Key Laboratory of Functional Molecular Imaging of Tumor (Shaoxing People’s Hospital, Shaoxing, Zhejiang, China), General Project of Zhejiang Province Health Science and Technology Plan (Grant Number: 2021KY1140), Medical and Health Science and Technology Platform Project of Zhejiang Province (Grant Number: 2018ZD047), General Project of Zhejiang Province Health Science and Technology Plan (Grant Number: 2022KY1296), General Project of Zhejiang Province Health Science and Technology Plan (Grant Number: 2022KY1291), and Medical and Health Science and Technology Plan Project of Zhejiang Province (Grant Number: 2020KY977). The funding sources had no role in the collection, analysis, or interpretation of the data or in the decision to submit the manuscript for publication.

## Conflict of interest

The authors declare that the research was conducted in the absence of any commercial or financial relationships that could be construed as a potential conflict of interest.

## Publisher’s note

All claims expressed in this article are solely those of the authors and do not necessarily represent those of their affiliated organizations, or those of the publisher, the editors and the reviewers. Any product that may be evaluated in this article, or claim that may be made by its manufacturer, is not guaranteed or endorsed by the publisher.
