# Supplementary material for: DCE-MRI radiomics models predicting the expression of radioresistant-related factors of LRP-1 and survivin in locally advanced rectal cancer
Source: Front Oncol. 2022 Aug 29;12:881341. doi: 10.3389/fonc.2022.881341 (PMC9465298; doi:10.3389/fonc.2022.881341)
Supplement: Supplementary file 1 [file Table_1.docx]

**Table 1.** Radiomics features derived from dynamic contrast-enhanced magnetic resonance imaging using OmniKinetics software

| Algorithms |  | | Radiomic features |
| --- | --- | --- | --- |
| First Order | | MinIntensity, MaxIntensity, MedianIntensity, MeanValue, stdDeviation, Variance, VolumeCount, VoxelValueSum, Root Mean Square, Range, MeanDeviation, RelativeDeviation, MinLocation, MaxLocation | |
| Histogram | | Energy, Entropy, Kurtosis, Skewness, Uniformity, FrequencySize, Uniformity Positive Pixel, Mean Positive Pixel, Quantile5, Quantile10, Quantile25, Quantile50, Quantile75, Quantile90, Quantile95 | |
| GLCM | | GlcmEnergy, GlcmEntropy, GlcmBinSize, GlcmTotalFrequency, GlcmMatrixMean, GlcmRelativeFrequency, Inertia, Correlation, InverseDifferenceMoment, ClusterShade, ClusterProminence, HaralickCorrelation, InvalidFeatureName | |
| Haralick | | AngularSecondMoment, Contrast, HaraVariance, sumAverage, sumVariance, sumEntropy, differenceVariance, differenceEntropy, inverseDifferenceMoment | |
| RLM | | MaxIntensity, MinIntensity, MinSize, NumberOfIntensityBins, MaxSize, NumberOfSizeBins, ShortRunEmphasis, LongRunEmphasis, GreyLevelNonuniformity, RunLengthNonuniformity, LowGreyLevelRunEmphasis, HighGreyLevelRunEmphasis, ShortRunLowGreyLevelEmphasis, ShortRunHighGreyLevelEmphasis, LongRunLowGreyLevelEmphasis, LongRunHighGreyLevelEmphasi | |
